# A study of the mechanism of small-molecule soybean-protein-derived peptide supplement to promote sleep in a mouse model

**DOI:** 10.1039/d0ra00389a

**Published:** 2020-03-18

**Authors:** Guofu Yi, Bushra Safdar, Yihao Zhang, You Li, Xinqi Liu

**Affiliations:** Beijing Advanced Innovation Center for Food Nutrition and Human Health, Beijing Engineering and Technology Research Center of Food Additives, Beijing Technology and Business University (BTBU) Beijing 100048 China li0187@163.com liuxinqi@btbu.edu.cn

## Abstract

Here, the effects of dietary supplementation with small-molecule soybean-protein-derived peptide (SBP) on sleep duration in mice are described. The amounts of the neurotransmitters tryptophan (Trp, W), 5-hydroxytryptamine (5-HTP), serotonin (5-HT) and melatonin (MT) were determined by using an ELISA kit. Compared with the control group, the group of mice given 0.65 g kg^−1^ SBP showed 59.21% prolonged sleep at the third day of administration and significantly increased MT levels, by 95.31%. Western blotting analysis of 0.65 g kg^−1^ SBP revealed the presence of tryptophan hydroxylase (THP) and serotonin-*N*-acetyltransferase (AANAT) proteins, which increased the release of MT and upregulated the MT1 and MT2 receptor activities to alleviate sleep deprivation. Interestingly, the introduction of 2.60 g kg^−1^ SBP doubled the 5-HT content in the brain and promoted an awake state. As a result, the produced 5-HT could not be converted into MT in large amounts, so the sleep duration was shorter than that of the control group. These findings suggested the potential of using SBP in appropriate amounts as functional ingredients in various food products to improve sleep in elderly people afflicted with sleep disorders.

## Abstract 2

Here, the effects of dietary supplementation with small-molecule soybean-protein-derived peptide (SBP) on sleep duration in mice are described.

## Graphical abstract

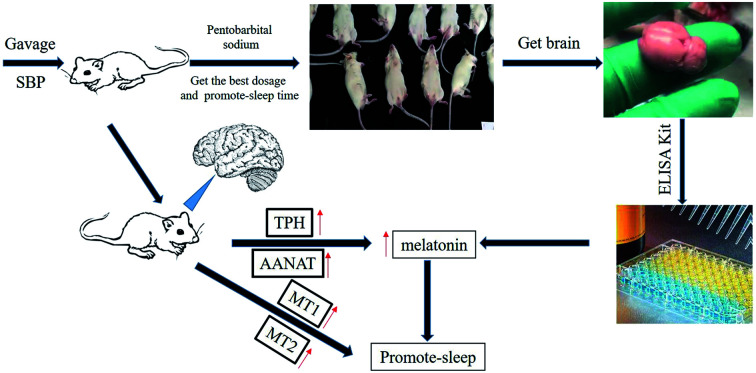

## 1 Introduction

**Fig. 1:**
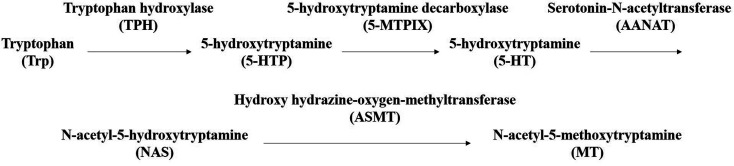
The synthetic route to MT.

Sleep is an active process of the human body that restores the spirit and relieves fatigue. Adequate sleep is one of the three health standards recognized by the international community.^1^ Nowadays, about a third of the people in the world have sleep problems. About 38% of the Chinese population displays various types of sleep disorders. Moreover, sleep disorders are 1.5–2 times more prevalent in women than in men. Elderly people are even more at risk for sleep disorders, which are found specifically in 40–70% of the elderly population.^2–4^ People with long-term sleep disorders suffer from a compromised quality of life such as cognitive impairment, memory loss, degraded ability to think, and delayed reaction ability. In severe cases, sleep disorders may even cause physical anxiety, depression, neurodegeneration and other mental illnesses.^5–8^ The occurrence of sleep has been reported to be closely related to the release of various neurotransmitters (interleukin IL-6, interleukin-1IL-1β, prostaglandins (PGD2, PGE2), tumor necrosis factor (TNF-α), neuropeptide substance P (SP), nitric oxide (NO), γ-aminobutyric acid (GABA), 5-hydroxytryptamine (5-HT), dopamine (DA), melatonin (MT), *etc.*) by the central nervous system and receptors.^9,10^ However, only MT and 5-HT levels significantly changed in experimental models, but the data did not show that other neurotransmitter changes were not statistically significant. 5-HT is an anthraquinone derivative originally found in serum and widely distributed in mammalian tissues. It is a neurohormone that has an important role in regulating the sleep/awake cycle, eating, mood control, and stress response.^11–14^ 5-HT in the blood cannot cross the blood–brain barrier; so in the brain, it is produced as a result of first the dehydroxylation of Trp by tryptophan hydroxylase (THP), which forms 5-hydroxytryptamine (5-HTP), and then the decarboxylation of 5-HTP by aromatic amino acid decarboxylase (5-MTPIX) to finally form the 5-HT.^15,16^ MT is then synthesized from 5-HT in two steps: 5-HT is first subjected to a reaction catalyzed by AANAT to form *N*-acetyl-5-hydroxytryptamine, which is then methylated using hydroxy hydrazine-oxygen-methyltransferase (ASMT) to obtain MT.^17,18^ The MT synthesis route is summarized in Fig. 1.

The level of MT secretion directly affects the length and depth of sleep, with insufficient secretion in the brain causing sleep disorders and disturbances in multiple systems.^19^ A light signal is transmitted to the suprachiasmatic nucleus in the brain through the retina, which regulates pineal secretion of MT. MT regulates the biological sleep-arousal rhythm by activating receptors.^20^ The secretion of MT is consistent with the light cycle and sleep biorhythm, with 2 to 3 times higher amounts of MT released at night than in the day. MT1 receptor aggregates in the central nervous system (CNS), thalamus nucleus and other parts, and its role is to regulate sleep. MT2 is involved in the circadian rhythm. A direct oral administration of MT can cure sleep disorders but displays large liver first-pass effects, short half-life, low bioavailability, and short duration of efficacy.^21^ Braam *et al.* reported good effects of taking MT for a few weeks, but low metabolism and accumulation of MT in the body resulting from long-term intake, making the circadian rhythm disappear and decreasing the MT efficacy.^22^ In addition, sleep disorder can be treated with other drugs such as benzodiazepines,^23^ ramelteon,^24^ agomelatine^25^ and so on. But overuse of the drugs can cause irreversible harm and there are withdrawal effects.^26–28^

Trp is a precursor of 5-HT and MT, and its intake can increase levels of 5-HT and MT secretion in the body.^16,29^ Trp is also an essential amino acid that cannot be synthesized in the human body and must be acquired from food. For the elderly, the activities in the body of enzymes such as pepsin and trypsin decrease to 30–50% of their normal activities,^30,31^ which reduces the digestion and absorption of proteins. Therefore, Trp efficacy could become insufficient to meet the needs of the body in older people^30^ so that the synthesis of MT decreases and leads to sleep disorders. SBP is hydrolyzed from soybean protein isolate (SPI), and display the same essential amino acid composition as does SPI but with better digestion and absorption rates and less antigenicity. Compared with free amino acids, SBP also displays the characteristics of faster absorption, lower energy-requirement barrier and higher carrier saturation rate.^32,33^

In the current study, a mouse model involving the administration of pentobarbital sodium was used to study the effect of SBP on duration of sleep of mice. The neurotransmitters and regulatory enzymes/receptors in the brains of the mice were examined using an ELISA kit and western blots to elucidate the SBP action mechanism. Determination of this mechanism could provide a theoretical basis for the application of SBP as a functional food ingredient in the food and pharmaceutical industries for the purpose of improving sleep.

## 2 Materials and methods

### 2.1 SBP and SPI preparation

**Fig. 2:**
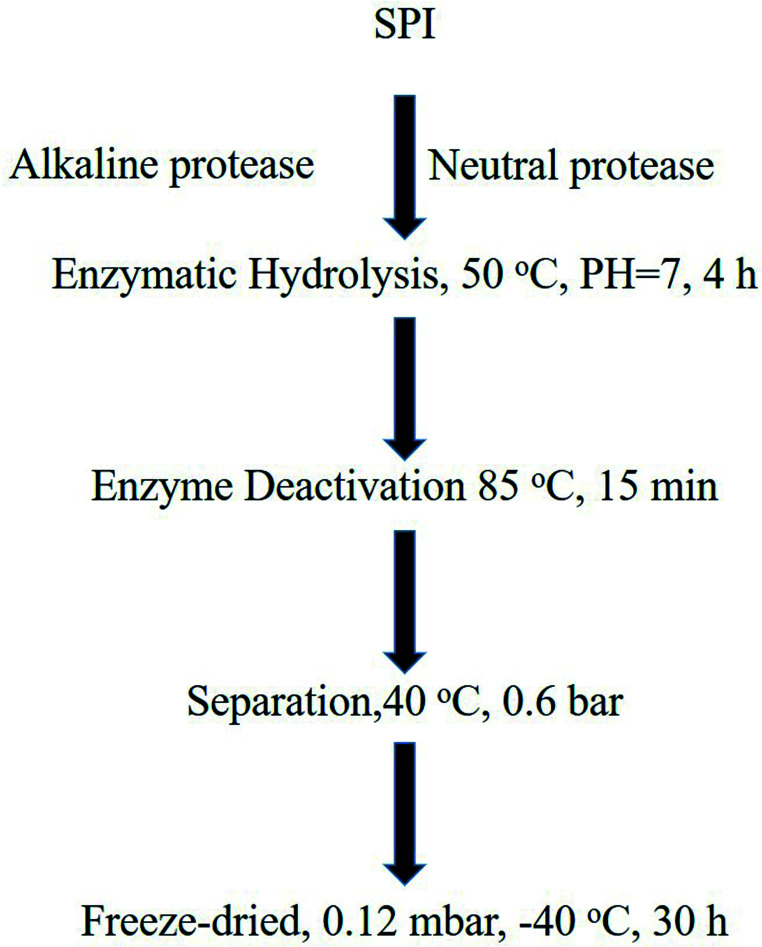
The preparation of peptides from SPI.

The SBP and SPI used in this study were provided by Nutrily Biotechnology, Ltd. (Anyang City, Henan Province, China, Patent No. CN107674900A) and were prepared according to the method described^34,35^ and as shown in Fig. 2. An ELISA kit, Trp, 5-HTP, 5-HT, and MT were purchased from Biologicals Novus (Litton, Colorado, USA). Antibodies against TPH (ab52954), AANAT (ab3505), MT1 (ab87639), MT2 (ab56308) were obtained from Abcam (Cambridge, UK).

### 2.2 Molecular weight distribution of SBP

The measurement of the molecular weight distribution of SBP was performed by using an LC20A (Shimadzu, JPN) gel filtration chromatograph. A TSK-GEL G2000SWXL column (5 μm, 7.8 × 300 mm) was used. The prepared peptide concentration was 1 μg mL^−1^, and all molecular weight standards (Gly–Gly–Gly, Gly–Tyr–Arg, bacitracin, insulin) were prepared at 1 mg mL^−1^. Before being injected into the column, both the sample and the standards were filtered through a 0.22 μm filter. The injection volume was 20 μL, the flow (water/acetonitrile/trifluoroacetic acid 80 : 20 : 0.1) rate was 0.5 mL min^−1^, and the detection wavelength was 220 nm.

### 2.3 Amino acid composition determination

SPI and SBP (1.00 g) were taken in two different hydrolysis tubes, respectively, to each of which a volume of 15 mL of a solution of 6 mol L^−1^ hydrochloric acid was added. Then the hydrolysis tubes were frozen for 3–5 min, subjected to a vacuum, and filled with nitrogen gas, over 3 cycles. The hydrolysis tubes were then sealed and left at 110 °C for 22 hours. The amino acid compositions of the resulting contents of the tubes were determined by using an amino acid analyzer (Biochrom 30+ amino acid analyzer, BioChrom Ltd) with a Na cation exchange column (8 μm, 4.6 × 200 mm), which was purchased from Waters Corporation (Milford, MA, USA), and the amino acids were derivatized with ninhydrin reagent after they were passed through the exchange column. The absorbance of the resulting material was measured at 440 nm (for proline) and 570 nm (for all other amino acids) and the results were expressed as milligrams per 100 g of sample powder.

### 2.4 Trp measurement

A mass of 100 mg of SPI and another mass of 100 mg of an SBP sample were weighed and placed into a polytetrafluoroethylene hydrolysis tube to which 4.0 mL of 4.0 mol L^−1^ of a lithium hydroxide solution was added. Then the polytetrafluoroethylene hydrolysis tube had its gaseous contents evacuated and was then filled with nitrogen for 3 min, sealed and had its contents subjected to hydrolysis at 110 ± 1 °C for 22 h. Then the mixture was taken out of the tube and cooled to room temperature. Its amino acid composition was determined by using an amino acid analyzer (Biochrom 30+ amino acid analyzer, BioChrom Ltd). Ninhydrin reagent was used for amino acid derivatization after the mixture was passed through a proteolysis separation column (4.6 mm × 200 mm), which was purchased from Waters Corporation (Milford, MA, USA). Absorbance of the sample was measured at a wavelength of 570 nm and results were expressed as milligrams per 100 g of sample powder.

### 2.5 Qualitative analysis of peptides by performing UHPLC-QE-MS

Five milligrams of peptide powder were weighed into EP tubes. After the addition of extracted solvent (acetonitrile–methanol–water, 2 : 2 : 1, containing internal 1 μg mL^−1^ standard), the samples were vortexed for 30 s, homogenized at 45 Hz for 4 min, and sonicated for 5 min in an ice-water bath. The quality control (QC) sample was prepared by mixing equal volumes of aliquots of the supernatants from all of the samples. LC-MS/MS analysis was performed by using an UHPLC system (1290, Agilent Technologies) with a UPLC HSS T3 column (2.1 mm × 100 mm, 1.8 μm) coupled to an Q Exactive (Orbitrap MS, Thermo) apparatus. The raw data were converted to mzXML format using ProteoWizard and processed by using MAPS software (version 1.0). The optimization conditions of the UHPLC-QE-MS mass spectrometer are shown in Table 1. After mass spectrometry scanning, Proteome Discoverer software (PD) (version 1.4.0.288, Thermo Fisher Scientific) was used to screen the mass spectra. After PD extraction, the spectra were examined using Mascot (version 2.3.2, Matrix Science). Peptide sequences were obtained after analysis of the Mascot search results and the first screening of the mass spectra.^36^

**Table 1:** The optimized conditions for UHPLC-QE-MS mass spectrometry

QE	Positive	Negative
Spray voltage (kV)	4.0 ESI^+^	3.6 ESI^−^
Capillary temperature (°C)	400	400
Sheath gas flow rate (Arb)	45	45
Aux gas flow rate (Arb)	15	15
Mass range (*m*/*z*)	70–3000	70–3000
Full mass resolution	70 000	70 000
MS/MS resolution	17 500	17 500
TopN	3	3
NCE/stepped NCE	20, 40, 60	20, 40, 60

### 2.6 Animal study

The entire animal study followed procedures that were consistent with the International Guiding Principles on Biomedical Research Involving Animals issued by the Council of the International Organization of Medical Sciences (CIOMS) and were approved by Peking University Third Hospital (IRB00006761-2018048). Six-week-old Wistar mice (20 ± 2 g) from the experimental animal center of Peking University were placed in cages at room temperature (23 ± 1 °C) and subjected to a 12 hour light/dark cycle with free access to food and water. Each mouse was used only once.

### 2.7 Sodium pentobarbital-induced sleep test

Based on the daily intake of 10 g of SBP by 70 kg human adults, the amounts of SBP fed to mice by weight were 1.30, 2.60 and 0.65 g kg^−1^ to conduct a concentration gradient study, and a 1.30 g kg^−1^ SPI group was used as a positive control group. The negative control group was fed with an equal volume of purified water. The mice were divided into 5 groups, each group consisting of 20 mice, and supplements were administered once a day for 9 consecutive days. Pentobarbital sodium (50 mg kg^−1^) was injected into the abdomen of each mouse 30 minutes after the end of each supplement administration, and the mice were laid on a 38 °C hot pad. Sleep latency was defined as the length of time from the injection of the pentobarbital sodium until the mouse was observed to not turn over for longer than 60 s. Sleep duration was defined as the length of time from when the mouse no longer turned over until the mouse started to turn over (*i.e.*, woke up).^37^

### 2.8 Brain neurotransmitter amounts determination

The mice were divided into 5 groups of 20 mice each, which were supplemented with 0.65, 1.30, and 2.60 g kg^−1^ of SBP, 1.30 g kg^−1^ of SPI, and an equal volume of purified water. Mice were fed once a day for 3 consecutive days. The mice were decapitated 30 min after the third day of supplementation and in each case the brain was quickly stripped and weighed, and then crushed in ice-cold phosphate buffer (PBS, pH 7.4, 10%, w/v) and centrifuged at 14 000 × *g* for 20 minutes. Supernatant was measured for the levels of the neurotransmitters Trp, 5-HTP, 5-HT and MT in the brain according to the instructions of the ELISA kit.

### 2.9 Western blot

The mouse brain tissue was lysed with ice-cold RIPA Lysis (Thermo Fisher Scientific, Shanghai, China), and incubated on ice for 30 min to extract proteins. The supernatant was collected from the product of centrifugation of the lysed brain tissue at 14 000 × *g* for 15 minutes at 4 °C for further testing. The protein concentration of each sample was measured by using a BCA Protein Assay Kit (Thermo Fisher Scientific, Shanghai, China). Then the supernatant was boiled in 5× loading buffer for 10 minutes, and electrophoresed on a 10% (w/v) sodium dodecyl sulfate-polyacrylamide gel (SDS-PAGE). The protein bands were transferred to an NC membrane (Millipore USA) and blocked with 5% (w/v) skim milk for 1 hour at room temperature. After being washed three times in Tris-buffered saline containing Tween 20 (TBST), the membranes were incubated overnight with designated primary antibodies (TPH, AANAT, MT1, MT2) at 4 °C, and were then washed again with TBST 5 times; and the secondary antibody conjugated with peroxidase was incubated at room temperature at a 1 : 5000 dilution. After washing the membrane, protein was detected using ECL (Millipore, USA) with GAPDH as a loading reference.

### 2.10 Data analysis

All experiments were repeated three times and the results were each expressed as mean ± standard deviation (SD). All data were analyzed by performing a one-way ANOVA using SPSS 16 software. Statistical significance was indicated by a *P* value less than 0.05.

## 3 Results and discussion

### 3.1 Determination of amino acid compositions of SPI and SBP

Between now and 2050, the demand for protein is expected to double, in part due to an expected gradual increase of the earth's population to approximately 9.5 billion, and the production of animal protein is hence expected to require more land and generate more greenhouse gases than the production of plant protein.^38^ Partial replacement of animal protein with plant protein, however, would have a positive effect on environmental protection.^39^ The Chinese diet includes a large amount of edible oil, which is obtained from the squeezing of soybeans. Most of the residue is used as animal feed. Extracting soybean protein from the leftover to obtain peptides could not only improve its utilization rate, but also make such protein less expensive, which would be of promotional value for the general public. Compared with other protein sources, soybeans grow over a wider area and are easier to obtain. Soybean provides a complete protein, with a full range of essential amino acids at appropriate relative amounts, and which is more suitable for absorption and utilization by the human body. Compared to most of other plant protein sources, soy is also rich in tryptophan (Trp).^40,41^

To assess the nutritional value of the peptides and verify the differences between SPI and SBP, the following amino acids were analyzed: aspartic acid, threonine, serine, glutamic acid, glycine, alanine, cysteine, valine, methionine, isoleucine, leucine, tyrosine, phenylalanine, lysine, histidine, arginine, proline, methionine and Trp. The results are shown in Table 2. Compared with the SPI sample, the SBP sample included more arginine, lysine and methionine, and less of the other amino acids. As shown in Table 1, the levels of Trp in the SPI and SBP samples were similar, so SBP can be used as a source of Trp supplementation. Since Trp is a precursor in the syntheses of 5-HT and MT, a reduction in Trp content in diet and nutritional supplements are likely to cause nutritional disorders in humans.^7^ Therefore, SBP are used as a supplemental source of Trp, and as a supply of an appropriate balance of amino acids, especially for adults with nutritional imbalances and for elderly showing reduced protease activity and resulting inadequate digestion.

**Table 2:** Amino acid compositions (%) of SPI and SBP^a^

Item	SPI	SBP
Aspartic acid	8.13 ± 0.60	2.67 ± 0.94
Threonine	1.50 ± 0.28	1.49 ± 0.26
Serine	3.84 ± 0.18	1.71 ± 0.03
Glutamic acid	16.22 ± 0.28	14.32 ± 0.64
Glycine	2.94 ± 0.11	1.70 ± 0.09
Alanine	2.67 ± 0.14	1.71 ± 0.11
Cysteine	0.68 ± 0.02	0.50 ± 0.02
Valine	4.14 ± 0.09	3.99 ± 0.18
Methionine	0.59 ± 0.11	0.66 ± 0.01
Isoleucine	3.29 ± 0.14	3.19 ± 0.19
Leucine	6.91 ± 0.20	6.14 ± 0.28
Tyrosine	2.77 ± 0.02	2.63 ± 0.06
Phenylalanine	4.45 ± 0.10	4.03 ± 0.16
Lysine	5.25 ± 0.07	5.59 ± 0.32
Histidine	2.07 ± 0.05	2.01 ± 0.12
Arginine	6.53 ± 0.06	6.65 ± 0.32
Proline	6.02 ± 0.05	5.89 ± 0.14
Methionine	1.21 ± 0.03	1.08 ± 0.06
Tryptophan	1.60 ± 0.02	1.49 ± 0.01

a Values are in mole% and expressed as the mean ± SEM.

### 3.2 SBP molecular weight distribution

**Fig. 3:**
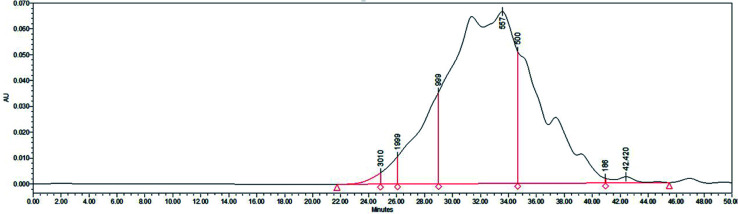
Molecular weight distribution of SBP.

Gel filtration chromatography was carried out using a Shimadzu LC20A instrument to determine the molecular weight distribution of SBP. As shown in Fig. 3, 86.33% of the SBP sample was composed of peptides with molecular weights between 186 and 1000 Da, and 11.86% of them showed molecular weights of 1000–2000 Da. The average molecular weight was determined to be about 721.57 Da, corresponding to about 3–6 amino acids.

### 3.3 Sequences of SBP

A mass spectrometry analysis indicated the presence of 1018 peptide types from 634 soybean proteins. All 61 of the Trp-containing peptide sequences are listed in Table 3. As shown in this table, leading razor protein tr|A0A0R0F1F3|A0A0R0F1F3_SOYBN included the most Trp-containing peptide fragments, specifically 11 different peptide chains. And the peptide chain SWGEDWGEIW contained the highest proportion of Trp, and the peptide RVDWKETPQAH in leading razor protein tr|I1L862|I1L862_SOYBN contained the least amount of Trp. In these peptides containing Trp, the carboxy terminus was found to be most often proline, followed by arginine, and then phenylalanine.

**Table 3:** Trp-containing peptides in an SBP sample

Sequence	Leading razor protein
DGWFR	tr|Q9S9D0|Q9S9D0_SOYBN
ALSWLR	tr|I1MWZ7|I1MWZ7_SOYBN
DGWFRL	tr|I1MKY2|I1MKY2_SOYBN
PNGPVWR	tr|B3TDK6|B3TDK6_SOYBN
RVDWKETP	tr|A0A0R0L186|A0A0R0L186_SOYBN
RDNPHWTS	tr|A0A0R0H569|A0A0R0H569_SOYBN
RVDWKETPQAH	tr|I1L862|I1L862_SOYBN
DAMDGWFR	tr|I1KRJ7|I1KRJ7_SOYBN
DKPNGPVW	tr|Q9FZP9|Q9FZP9_SOYBN
LSWLRLS	tr|I1LHP6|I1LHP6_SOYBN
EWPRKEE	tr|K7MPA8|K7MPA8_SOYBN
TLPFPWLPE	tr|Q9XET1|Q9XET1_SOYBN
QEWPRKE	tr|A9YT06|A9YT06_SOYBN
NGPVWRIS	tr|A0A0R0GA14|A0A0R0GA14_SOYBN
EQEWPRKEE	tr|A0A0R0KK84|A0A0R0KK84_SOYBN
FSWNVLQAA	tr|I1LWR7|I1LWR7_SOYBN
REEWPR, HEWHR, GPVWRIS, NPEEIPWGS, NPEEIPWGE, NGPVWRIS, PALSWLRL, SWGEDWGEIW, QEWPRK, WRSKKTQ, VWFPQPWR	tr|A0A0R0F1F3|A0A0R0F1F3_SOYBN
WELPDD, LSWLRL, EQEWPRKEE, DKPNGPVWR, QTFLWGRY, NGPVWRIS, ALSWLRLS	tr|A0A0R0KKD6|A0A0R0KKD6_SOYBN
GPVWR, WREDIRVA, DWKETP, WEEPFGP	tr|Q588Z6|Q588Z6_SOYBN
WDSIPI, KNPIGW, KNSWGEDWG	tr|C6T1V2|C6T1V2_SOYBN
NGPVWRIS, DFPALSWL	tr|I1M005|I1M005_SOYBN
SGDVWFP, DWVSLPGV, DVWDPFH, EIPWGST, MDGWFRL, LSWLRLS, KPNGPVWR	tr|O22120|O22120_SOYBN
NWKPGDP, DPDWYY	tr|O64458|O64458_SOYBN
REEQEWPR, PWGSTG, MDGWFR	tr|I1KC69|I1KC69_SOYBN
DKWHRVE, NGPVWRIS, GHPEWEL, DFPALWLL, DDRWHRVE	sp|P01070|ITRA_SOYBN

### 3.4 Sodium pentobarbital-induced sleep time test

**Fig. 4:**
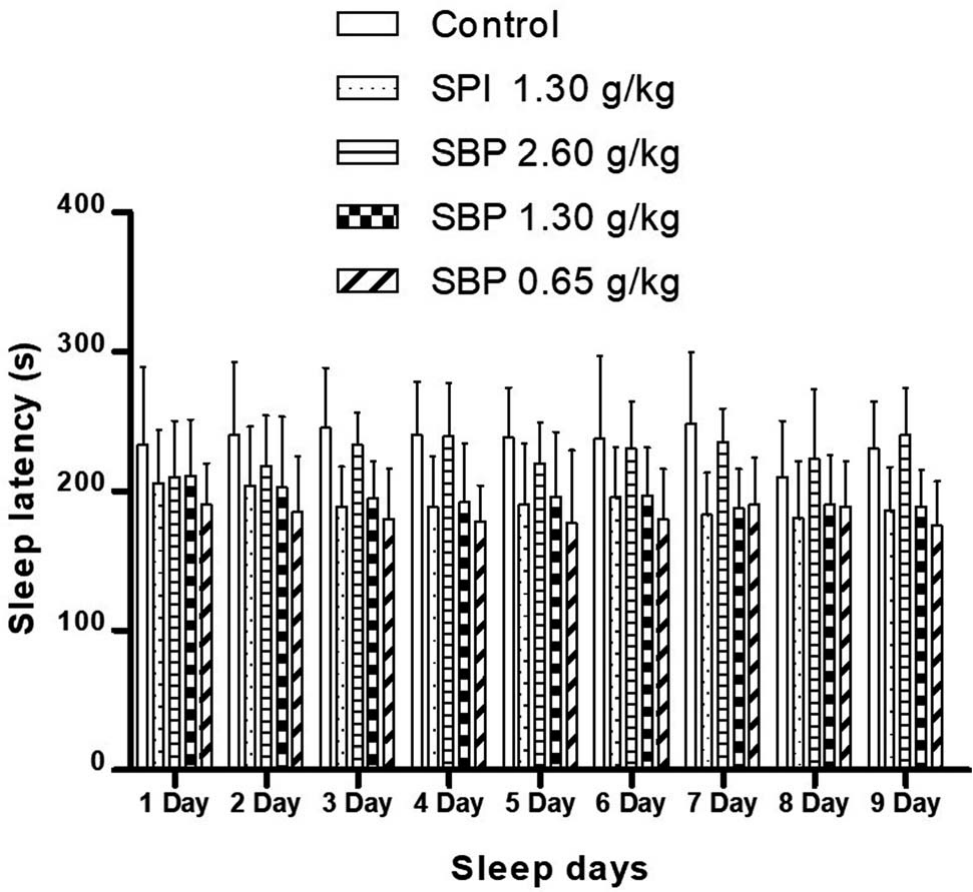
Sleep latency in mice with different SPI/SBP administration patterns. Data are presented as the mean ± SD. *P* < 0.05 = “*”, *P* < 0.01 = “**”, *P* < 0.001 = “***” *vs.* control group, *n* = 3 data points.

**Fig. 5:**
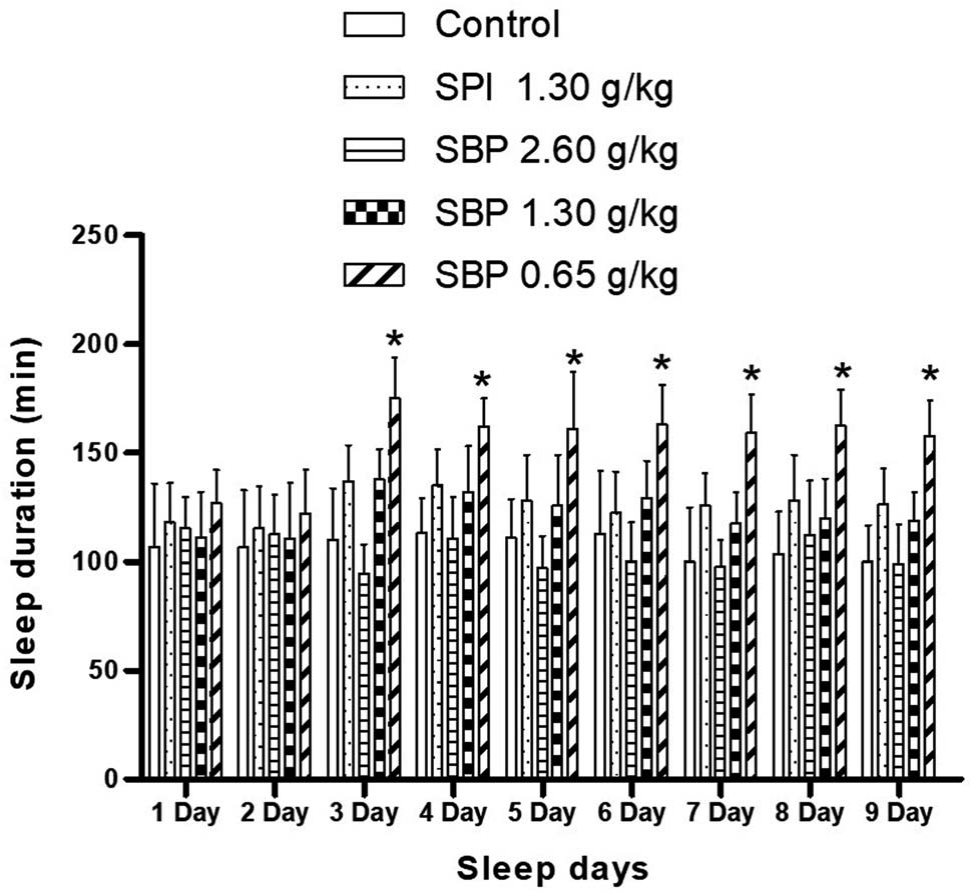
Sleep duration in mice with different SPI/SBP administration patterns. Data are presented as the mean ± SD. *P* < 0.05 = “*”, *P* < 0.01 = “**”, *P* < 0.001 = “***” *vs.* control group, *n* = 3 data points.

As shown in Fig. 4, mice showed a loss of torsional reflex within 6 minutes of being administered sodium pentobarbital (50 mg kg^−1^). Compared with the control group, the shortened latencies of the three above-described SBP groups were different. The 0.65 g kg^−1^ SBP group showed shorter latencies than the 2.60 g kg^−1^ SBP group, showing negative correlation between the dosage and effects. The 1.30 g kg^−1^ SPI group showed shorter latencies than the equivalent dose of the SBP group. There was a cumulative effect on sleep latency for each group except for the 2.60 g kg^−1^ SBP group over the 9 day administration, but no case showed statistical significance. As shown in Fig. 5, during the 9 day dosing process, the SBP dosage of 0.65 g kg^−1^ started to have an effect on prolonging sleep time on the third day and presented the best effect. Compared with the dosage of the control group, the SBP dosage of 0.65 g kg^−1^ significantly (*P* < 0.05) prolonged sleep duration, by 59.21%, consistent with the reason that SBP is used in regulating sleep. Peptides are digested into amino acids or smaller peptide chains that can be absorbed. In contrast to the fast action of drugs, the SBP dosage of 0.65 g kg^−1^ was effective only starting on the third day. Compared with the control group, the SBP dosage of 2.60 g kg^−1^ did not increase sleep duration, but instead had a tendency to decrease sleep duration, albeit with no statistical significance. Determining the reason for the decrease in the duration of sleep with increasing dosage requires further study.

### 3.5 Determination of levels of Trp, 5-HTP, 5-HT and MT in the brain

**Fig. 6:**
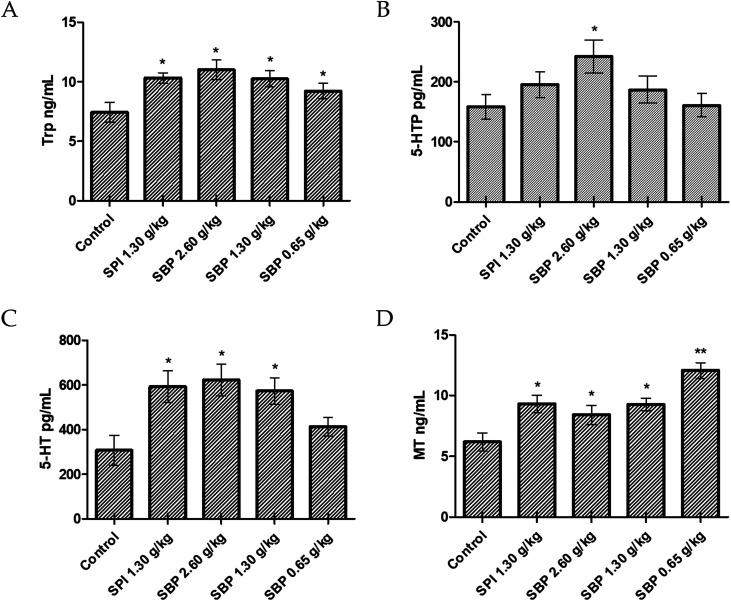
Concentrations of Trp (A), 5-HTP (B), 5-HT (C), and MT (D) in the brains of tested mice. Data are presented as the mean ± SD. *P* < 0.05 = “*”, *P* < 0.01 = “**”, *P* < 0.001 = “***” *vs.* control group, *n* = 3 data points.

Compared with the control group, the 2.60 g kg^−1^ SBP group showed a significantly greater (*P* < 0.05) amount of Trp in the brain, specifically 71.42% more Trp (Fig. 6A). As the SBP dosage was increased, the amount of Trp in the mouse brain also increased, showing a concentration dependence. As shown in Fig. 6B and C, the SBP dosage of 2.60 g kg^−1^ yielded a significantly increased level of 5-HT (*P* < 0.05) in the mouse brain, specifically by 43.75%, and significantly increased level of 5-HTP (*P* < 0.05), by 90.61%, compared with the control group. The amounts of Trp and 5-HT in the brains of the mice increased as the SBP level was increased. However, as shown in Fig. 6D, compared to the control group, the 2.60 g kg^−1^ SBP group showed increased MT content in the brain, and the MT level was not significantly different from the SPI and 1.30 g kg^−1^ SBP groups. As shown in Fig. 5, compared with the control group, the prolongation of sleep on the third day was decreased for the 2.60 g kg^−1^ SBP group. Melancon and colleagues reported that 5-HT released in the brain helped to promote sleep. 5-HT was released more during the day than at night. Trp supplementation in the diet can stimulate 5-HT release and promote sleep. However, 5-HT released in the diencephalon and cerebrum might inhibit supraspinal neural networks and cause one to wake up.^42^ Ito *et al.* reported that drugs (such as SSRI) that increased the levels of 5-HT in the brain generally caused humans and rodents to wake up from sleep. Doubling the amount of 5-HT was observed to lower the threshold of sleep arousal and disrupt the sleep NREM period, leading to waking up from sleep.^43^ In addition, microdialysis studies showed that 5-HT levels in the brain were highest when waking up, lower during no rapid eye movement (NREM) and lowest during rapid eye movement (REM). Therefore, an increase in the level of 5-HT can improve the ability to wake up.^44^ Administration of 2.60 g kg^−1^ of SBP nearly doubled the amount of 5-HT in the mouse brain, which could be the reason that the sleep duration was less for this group than for the control group (Fig. 5). Because MT is a sleep-promoting neurotransmitter, one that in particular increases NREM and REM during sleep, SBP at 2.60 g kg^−1^ could make the 5-HT content in the brains of mice too high to access NREM sleep and lead to decreased synthesis of MT.

Compared to the control group, the 0.65 g kg^−1^ SBP group showed 95.31% more MT (Fig. 6D), and higher (albeit statistically insignificantly higher) levels of release of 5-HT (Fig. 6C). However, as shown in Figure 5, 0.65 g kg^−1^ had the most significant effect on prolonging sleep on the third day of administration. Therefore, the prolonging of the sleep of the tested mice might have been caused by increased MT contents in their brains. As shown in Fig. 6C and D, compared with the control group, the 1.30 g kg^−1^ SPI and 1.30 g kg^−1^ SBP groups showed significantly increased levels of 5-HT and MT (*P* < 0.05). Since too much 5-HT could cause one to wake up, and since elevated MT promotes sleep, the effects of 5-HT and MT could cancel each other out. Therefore 1.30 g kg^−1^ of SPI and 1.30 g kg^−1^ of SBP showed no significant difference in prolonging sleep in mice (Fig. 5).

### 3.6 Regulation of TPH protein expression by secreted 5-HT

**Fig. 7:**
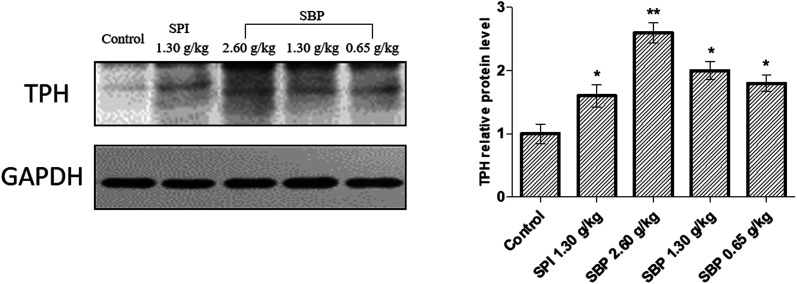
The effects of SBP on the expression of TPH. Data on the right are expressed as mean ± SD (*n* = 3). Data are presented as the mean ± SD. *P* < 0.05 = “*”, *P* < 0.01 = “**”, *P* < 0.001 = “***” *vs.* control group, *n* = 3 data points.

To further elucidate the mechanism of 5-HT regulation of the mouse brain neurotransmitter by SBP, we studied TPH, the key rate-limiting enzyme for the synthesis of 5-HT, and extracted brain proteins for western blot analysis, as shown in Fig. 7. Compared with the control group, the protein expression of TPH was significantly (*P* < 0.01) upregulated for the 2.60 g kg^−1^ SBP group, which might be the reason that the administration of SBP at 2.60 g kg^−1^ increased the levels of 5-HT in the mouse brain. The three SBP dosage groups of mice showed a concentration-dependent expression of TPH protein in their brains. Both the SBP (*P* < 0.05) and SPI (*P* < 0.05) groups showed significantly increased protein expression of TPH, but the same administration level showed no significant difference in TPH expression between the SPI and SBP groups.

### 3.7 Effect of SBP on AANAT, MT1, MT2 protein expression

**Fig. 8:**
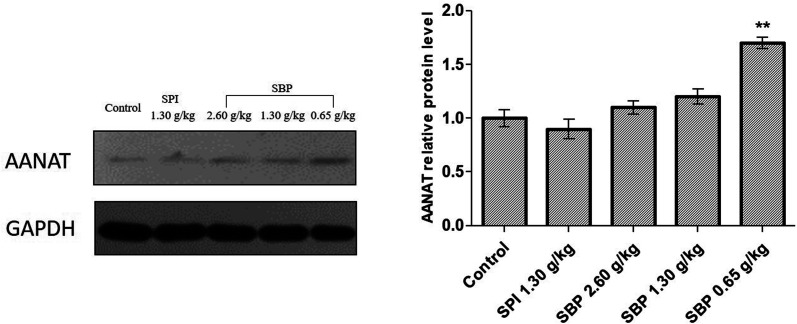
The effects of SBP on the expression of AANAT. Data on the left are expressed as mean ± SD (*n* = 3). Data are presented as the mean ± SD. *P* < 0.05 = “*”, *P* < 0.01 = “**”, *P* < 0.001 = “***” *vs.* control group, *n* = 3 data points.

As shown in Fig. 8, the protein expression of AANAT was significantly upregulated in the 0.65 g kg^−1^ SBP group (*P* < 0.01) compared to the control group, but not so in the 2.60 g kg^−1^ and 1.30 g kg^−1^ SBP groups. The expression of AANAT protein in the SPI group was not statistically significantly different than that in the control group. It was reported that increased AANAT activity could decrease 5-HT levels and increase MT levels,^45^ which could have caused the 5-HT content in the brains of the 0.65 g kg^−1^ SBP group mice to not be as high as in the other SBP treatment groups (Fig. 6C) due to its enhancement of AANAT expression. That is, more of the 5-HT was converted into MT.

**Fig. 9:**
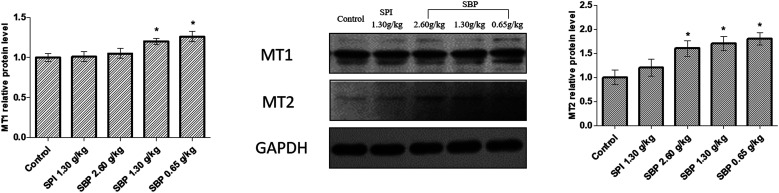
The effects of SBP on the expression of MT1 and MT2. Data are expressed as mean ± SD (*n* = 3). Data are presented as the mean ± SD. *P* < 0.05 = “*”, *P* < 0.01 = “**”, *P* < 0.001 = “***” *vs.* control group, *n* = 3 data points.

MT is formed in the pineal gland in a light-regulated manner as a result of enzymatic conversion from 5-HT, and regulates sleep by activating two high-affinity G protein-coupled receptors, namely MT1 and MT2.^46^ We therefore studied the protein expression of the MT receptors MT1 and MT2. As shown in Fig. 9, the protein expression of MT1 was significantly upregulated in the 0.65 g kg^−1^ SBP group (*P* < 0.05) compared to the control group. However, the MT1 expression was not significantly increased in the SPI and 2.60 g kg^−1^ SBP groups. Compared with the control group, all three SBP groups showed significantly upregulated MT2 protein expression levels, while the 1.30 g kg^−1^ SPI group did not show any significantly different MT2 expression.

TPH and AANAT are two rate-limiting enzymes that synthesize MT in brains.^47^ Therefore, the mechanism by which 0.65 g kg^−1^ SBP prolong sleep could involve increasing the activities of the MT1 and MT2 receptors and the Trp content in the brain to enhance the TPH (Fig. 7) and AANAT activities (Fig. 8) so that a large amount of Trp might be converted into 5-HT and in turn be converted into MT in large quantities (Fig. 6D).

There are also certain limitations in this study. The SBP were not further separated and purified. The mixture obtained from the enzymatic hydrolysis of SPI was composed of many peptide fragments, some of which have good bioavailability and lose this biological activity after purification. In addition, from a macro-nutritional point of view, a good balance of essential amino acids and a fast absorption rate are key factors for rapidly providing protein to the body. However, certain single peptides may not yield a good balance of amino acids. Therefore, for people with a nutritional imbalance, the collective effect from various peptide chains could be better than that from a single biologically active peptide. A mixture of peptide chains is used to verify that the test is closer to real life than testing with a single peptide chain. In our previous research, a mixture of peptides was used to supplement nutrition and regulate the healing and inflammation of burned mice with significant success,^34^ showing broad implications for treating other medical conditions. Furthermore, in the experiments on the sleep duration of mice, only pentobarbital sodium induction was used, and not the more advanced polysomnography (PSG), due to the limitations of funding and the experimental site. Since the study showed a significant effect of 0.65 g kg^−1^ SBP on sleep induction in mice, future experiments may consider adjusting the measurement range to investigate the low dosage effects of SBP as well as SPI on sleep. Finally, the release of neurotransmitters was detected by using an ELISA kit instead of a current advanced microdialysis instrument, which can be utilized to track continuous release from mouse brains during the day and night.

## 4 Conclusions

The current results showed that low-molecular-weight peptides (186–1000 Da) derived from SPI can effectively provide a supplement of Trp. By regulating the activities of the rate-limiting enzymes TPH and AANAT, the content of MT in the brain can be increased, and the activities of the receptors MT1 and MT2 can be extended to prolong sleep. For those whose nutrition is poor due to a lack of MT or the insufficient secretion of digestive enzymes and nutritional imbalance, especially those who have an insufficient intake of the essential amino acid Trp, SBP can be used as a potential food component. According to the results of this study, using SBP supplements at too high of a level can cause the excessive secretion of 5-HT without prolonged sleep. Instead a dosage level of 0.65 g kg^−1^ was concluded from the results to be appropriate.

## Abbreviations

The following abbreviations are used in this manuscript:

SBP: Small-molecule soybean protein-derived peptide
Trp, W: Tryptophan
5-HTP: 5-Hydroxytryptamine
5-HT: Serotonin
MT: Melatonin
THP: Tryptophan hydroxylase
AANAT: Serotonin-*N*-acetyltransferase
5-MTPIX: Aromatic amino acid decarboxylase
ASMT: Hydrazine-oxygen-methyltransferase
NREM: No rapid eye movement
REM: Rapid eye movement

## Author contributions

Conceptualization, G. F. Y. and Y. L.; methodology, G. F. Y., S. B., Y. H. Z., Y. L. and X. Q. L.; software, S. B. and Y. H. Z.; validation, Y. L., X. Q. L. and S. B.; formal analysis, G. F. Y.; investigation, X. Q. L.; writing and editing the manuscript, G. F. Y., Y. L., S. B. and Y. H. Z. project administration, X. Q. L.; and funding acquisition, X. Q. L. All authors have read and agreed to the published version of the manuscript.

## Conflicts of interest

The authors declare no competing financial interests.

## Supplementary Material
